# Design of an Ultrasound-Navigated Prostate Cancer Biopsy System for Nationwide Implementation in Senegal

**DOI:** 10.3390/jimaging7080154

**Published:** 2021-08-20

**Authors:** Gabor Fichtinger, Parvin Mousavi, Tamas Ungi, Aaron Fenster, Purang Abolmaesumi, Gernot Kronreif, Juan Ruiz-Alzola, Alain Ndoye, Babacar Diao, Ron Kikinis

**Affiliations:** 1 School of Computing, Queen’s University, Kingston, ON K7L 2N8, Canada; mousavi@queensu.ca (P.M.); ungi@queensu.ca (T.U.); 2 Department of Medical Biophysics, Schulich School of Medicine & Dentistry, Western University, London, ON N6A 5B7, Canada; afenster@robarts.ca; 3 Department of Electrical and Computer Engineering, University of British Columbia, Vancouver, BC V6T 1Z4, Canada; purang@ece.ubc.ca; 4 Austrian Center for Medical Innovation and Technology, 2700 Wiener Neustadt, Austria; gernot.kronreif@acmit.at; 5 Departamento de Señales y Comunicaciones, University of Las Palmas de Gran Canaria, 35001 Las Palmas, Spain; juan.ruiz@ulpgc.es; 6 Department of Urology, Hôpital Aristide Le Dantec, Cheikh Anta Diop University, Dakar 10700, Senegal; alain.ndoye@ucad.edu.sn (A.N.); babacar.diao@ucad.edu.sn (B.D.); 7 Department of Urology, Ouakam Military Hospital, Dakar BP 5321, Senegal; 8 Harvard Medical School, Brigham and Women’s Hospital, Boston, MA 02115, USA; kikinis@bwh.harvard.edu

**Keywords:** prostate cancer, biopsy, ultrasound, Africa, frugal technology

## Abstract

This paper presents the design of NaviPBx, an ultrasound-navigated prostate cancer biopsy system. NaviPBx is designed to support an affordable and sustainable national healthcare program in Senegal. It uses spatiotemporal navigation and multiparametric transrectal ultrasound to guide biopsies. NaviPBx integrates concepts and methods that have been independently validated previously in clinical feasibility studies and deploys them together in a practical prostate cancer biopsy system. NaviPBx is based entirely on free open-source software and will be shared as a free open-source program with no restriction on its use. NaviPBx is set to be deployed and sustained nationwide through the Senegalese Military Health Service. This paper reports on the results of the design process of NaviPBx. Our approach concentrates on “frugal technology”, intended to be affordable for low–middle income (LMIC) countries. Our project promises the wide-scale application of prostate biopsy and will foster time-efficient development and programmatic implementation of ultrasound-guided diagnostic and therapeutic interventions in Senegal and beyond.

## 1. Introduction

Prostate cancer (PCa) is the most frequent cancer and the leading cancer-related cause of death in African men [1]. Early-stage PCa is often asymptomatic or shares symptoms of benign prostatic hyperplasia. Due to the lack of screening and public awareness of prostate diseases in Africa, PCa is discovered at advanced stages [2]. In Senegal, of over 60% of patients affected with advanced cancer, only less than 40% are a treatable form of localized disease [3,4], a stark contrast to the USA where about 89% of PCa cases are diagnosed early [5]. Alas, the 5-year survival rate after diagnosis hovers around 30% in Senegal [4], versus 98% in the USA [5].

Once diagnosed, early stage PCa is a treatable disease. The primary treatment option is total surgical resection via radical prostatectomy, which is available in Senegal. The ultimate key to PCa diagnosis is core tissue biopsy (PBx). This procedure has evolved since the early 1990s and is well standardized [6]. PBx is usually performed transrectally under local anesthesia, with transrectal ultrasound (TRUS) imaging guidance, and it is well tolerated [7,8]. Bleeding is the most frequent side effect, usually resolved naturally while infection is suppressed with antimicrobial prophylaxis [9]. In addition to cancer detection, prostate biopsy plays a crucial role in grading the disease and making treatment decisions. While PBx is universally available in North America, in Senegal it is only offered in a small number of hospitals in the capital, Dakar, where trained urologists and pathologists, and the requisite equipment, are available. Of the 16 million inhabitants of Senegal, about 3.5 million people living in the Dakar metropolitan area have “geographical access” to advanced urological services. In the Dakar region however, many patients still cannot get a prostate biopsy, because the waiting lists are too long in the public clinics and the procedure is prohibitively expensive in the private clinics.

Transrectal ultrasound-guided PBx is difficult to perform; simultaneous imaging, anatomical interpretation and placement of the biopsy needle requires extensive training and experience. Ultrasound offers poor imaging of the prostate anatomy zones and low sensitivity in showing PCa foci growing in these zones. Altogether, standard PBx suffers from a low diagnostic yield (~40%), high false negative rates [7,10], and cancer lesions as large as half a sugar cube (0.5 cc, the smallest clinically significant prostate cancer [11]), are often missed. The outcomes are worse in Senegal, where it is reported that among all patients referred for PBx, cancer was found in only 30%, even though over 60% of Senegalese patients have advanced disease [12], showing the need for improvements in how the biopsy procedure is delivered in Senegal. In high-income countries, “fusion biopsy”, also known as “real-time virtual sonography” (i.e., spatial navigation that registers ultrasound with multi-parametric MRI), has emerged as a method for improving prostate biopsy [13,14]. This approach is not feasible in West Africa, where fewer than 40 high-field MRI units serve a population larger than North America, with Senegal having just 2 suitable MRI units [15]. There is a critical need to address the lack of availability and the quality of prostate cancer biopsy in Senegal, with high reward from doing so: the ability to diagnose prostate cancer early will shift clinical practice and allow us to cure many patients who will eventually succumb to terminal cancer. 

Our objective is to create a prostate biopsy system, herein named NaviPBx, that visualizes cancer-suspicious locations highlighted in multiparametric transrectal ultrasound imaging, and allows for the real-time navigation of the biopsy needle to ensure the accurate targeting of potential lesions, and the extensive evaluation of its feasibility, usability and scalability. The essence of our innovation lies in combining low cost with functionality and usability, optimized for conditions in Senegal, yielding a system with functionality and technical performance comparable with state-of-the-art fusion biopsy systems, at a fraction of the cost. Our technical approach concentrates on “frugal technology”, intended to be affordable in Senegal and generally for low–middle income (LMIC) countries.

We will integrate techniques, the clinical effectiveness of which have been extensively validated in peer-reviewed literature, resulting in a system that promises to enable the first national-level implementation of an image-guided intervention program based entirely on free, open-source software. NaviPBx is set to be implemented nationwide through the Senegalese Military Health Service, in a total of ten hospitals across the country, in which over 90% of the patients are civilians with no connection to the military.

## 2. System Design and Architecture

### 2.1. System Design

We aim to create a prostate biopsy system that visualizes cancer-suspicious target locations highlighted in multiparametric transrectal ultrasound imaging, and facilitates real-time navigation of the biopsy needle to allow for the systematic and targeted sampling of possible prostate cancer locations. We derive the proposed system from the current most advanced implementation of PBx, “fusion biopsy” (a.k.a. “real-time virtual sonography”), which entails the registration of MRI and TRUS [13,14]. The main advantages of MRI lie in two key functions for determining and accessing the biopsy targets: (1) showing the intraprostatic anatomy for systematic sampling and (2) showing cancer-suspicious locations for additional sampling. The real-time tracking of the TRUS probe enables the fusion action, allowing the physician to navigate the biopsy needle to the desired biopsy target locations without having to mentally interpret low-quality live 2D TRUS images. As MRI is scarcely available in Senegal, our research strategy is to develop a PBx system that provides most of the benefits of fusion biopsy without using MRI, while retaining the same physical layout, anatomical access, and overall workflow. We will use spatially navigated, multiparametric, transrectal ultrasound imaging to solve this problem. In doing so, we will adopt methods whose clinical utility have been extensively evaluated in the peer-reviewed literature. 

As envisioned, NaviPBX will have about the same overall functionality as other commercial fusion biopsy systems (e.g., UroNav), except in NaviPBX the pre-operative multi-parametric MRI imaging will be replaced with multi-parametric ultrasound imaging. Unfortunately, ultrasound will no doubt be inferior to MRI, but it is our only viable prostate imaging option in Senegal. Additionally, NaviPBX will not involve a separate MR imaging session. This is a significant disadvantage in Senegal, where deep-routed sociocultural reflexes hinder public discourse about genitourinary diseases and, as a result, discourage men from seeking treatment for prostate disease.

**Figure 1:**
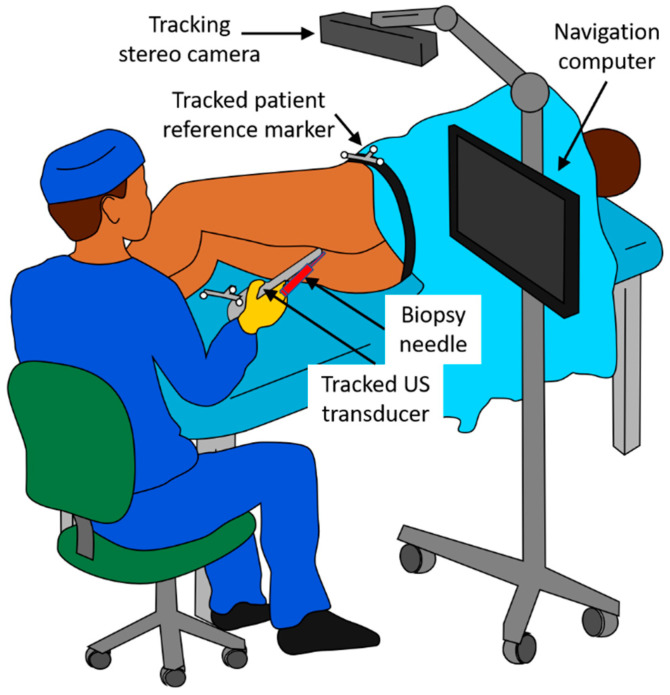
Physical layout of the NaviPBx prostate biopsy system, as envisioned.

NaviPBx is envisioned as shown in Figure 1. The patient is in the lateral decubitus position, slightly pulled up with their knees bent and rectum cleansed, the most common approach to PBx. (The alternative would be transperineal PBx performed under full or lumbar anesthesia with extensive special hardware, altogether impractical). A sterile latex cover filled with coupling gel is used for the TRUS probe. A standard sterile biopsy needle guide is clipped on the TRUS probe. An optical stereo tracking camera is mounted over the scene. Optical tracking has been used reliably for decades in surgical navigation systems, which are constantly improving and becoming less expensive. Optical tracking markers are reusable and inexpensive to manufacture locally. (The alternative would be electromagnetic (EM) tracking, used in the current commercial fusion biopsy systems. While EM does not require a clear line of sight, it has numerous drawbacks. It is adversely affected by metal objects and electric currents and is expensive and difficult to resupply in Senegal. Current EM tracking systems are produced by one company monopolizing the market, having kept both the price and technology stagnant for over a decade). Standard optical markers are clipped onto the transducer and attached onto the patient’s hip, allowing the live TRUS image to be tracked with respect to the patient. There is an un-obstructed line of sight for the tracking camera; no drapes, tools or persons obstruct the view. The biopsy needle guide and the tracking markers are attached to the TRUS transducer reproducibly and always in the same position. The transducer is delicately inserted into the rectum, rotated, and translated while observing the ultrasound image on the screen.

According to our design, the physician is assisted by two key system functions in determining the biopsy target positions. Firstly, to observe the intraprostatic anatomy for systematic sampling, the physician begins with an exploratory TRUS scan of the prostate. NaviPBx records the images and compounds a 3D TRUS volume. It is worth noting that 3D TRUS is used in the same manner in all fusion biopsy systems, e.g., Philips UroNav, Eigen Artemis™, and KOELIS Trinity^®^, and thoroughly validated in the peer-reviewed clinical literature. NaviPBx automatically segments the prostate gland and computes a map of the intraprostatic McNeal zonal anatomy [16], based on which it computes the systematic target locations (typically 12 cores), according to convention [7]. A standard biopsy needle is inserted into the guide channel for harvesting the samples. The physician manipulates the transducer in the rectum, assisted by real-time “labels” painted over the live 2D TRUS image, e.g., current biopsy needle path, prostate gland, McNeal zonal anatomy map, and the preplanned systematic biopsy locations (Figure 1). As the transducer displaces and deforms the prostate gland, NaviPBx automatically adjusts the labels on the live 2D TRUS image. Secondly, to show the cancer-suspicious locations for additional sampling, multiparametric transrectal ultrasound (mpTRUS) imagery is constructed from multiple ultrasound modalities (B-mode, elasticity, and Doppler). In the peer-reviewed literature, each of these modalities has been proven to contribute to recognizing PCa. With the use of 3D tracking, NaviPBx constructs a hyperdimensional image array from the available component ultrasound modalities, allowing for the analysis and visualization of them together in 3D. Finally, NaviPBx provides a variety of measurement tools to complete the diagnostic picture: dimensions, volume, shape and symmetry of the prostate; integrity of the prostate capsule; echogenicity of each McNeal zone; presentation (dimensions, symmetry, and angulation) of the seminal vesicles; presentation of the ampullae of the ducti deferens; stiffened areas quantified in elasticity imaging; and vascularization measured in Doppler imaging. We plan for these measurements to be made offline, conveniently after the biopsy in the compounded mpTRUS volume.

### 2.2. System Architecture

**Figure 2:**
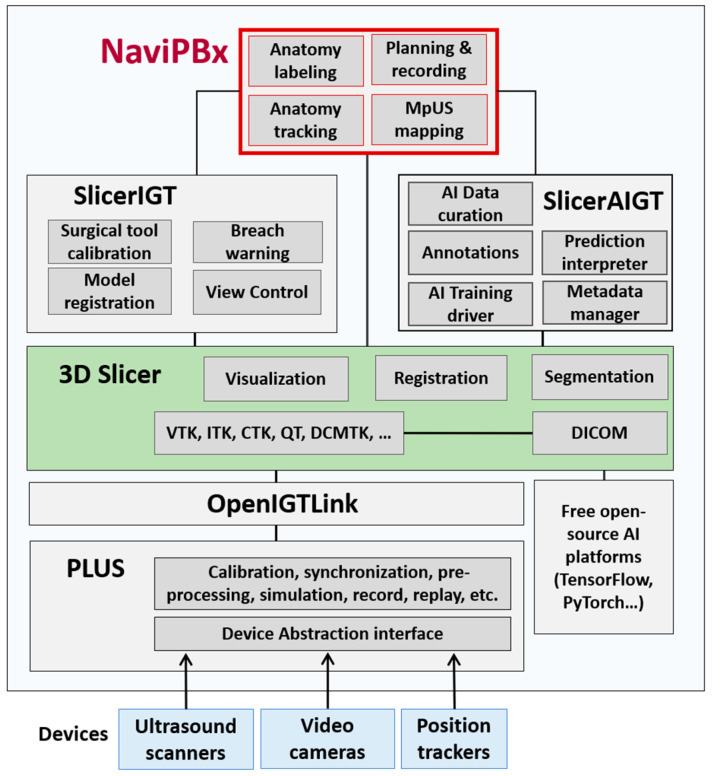
Software system architecture for the NaviPBx prostate biopsy system.

NaviPBx (Figure 2) builds from the software 3D Slicer (www.slicer.org (accessed on 8 August 2021)), a free, open-source software ecosystem for multimodal medical image analysis and visualization, simultaneously extended for image-guided interventions [17].

The software, 3D Slicer, facilitates the exploration, evaluation and clinical translation of novel methods by freeing researchers from recurring tasks. The software offers thousands of features through 81 core modules and 156 extension packages. It follows a modular design, where each module is a feature-complete functional unit. Generic functions (e.g., image import/export, segmentation, and registration) used in most clinical applications are grouped in the 3D Slicer core. Domain-specific features, such as image-guided therapy and interventions, are packaged in dynamically loadable extensions, allowing users to “cherry pick” features from multiple extensions, without programming overhead. The extensions of 3D Slicer, and the software itself, run on all three major operating systems without hardware dependency. Developed since 1998 by a worldwide developer community with over 2000 years of FTE effort, 3D Slicer has become the leading open-source software resource in its field. Cumulative downloads of 3D Slicer resources have surpassed one million, and its current download rate is 400 times per day.

The Public Library for Ultrasound Toolkit (PLUS, www.PlusToolkit.org (accessed on 8 August 2021)) [18] is an abstraction layer for hardware devices, e.g., ultrasound scanners, position tracking devices, cameras, and generally any kind of data-streaming device. PLUS provides generic functions for pre-processing, such as the 3D ultrasound volume compounding [19] and live volume rendering [20] of tracked ultrasound image streams. PLUS offers complete end-user software applications for spatial and temporal calibration, an essential toolset in clinical translation and system maintenance. PLUS allows for seamlessly changing hardware devices without any change to the rest of the system. Additionally, PLUS supports the simultaneous running of multiple data streamers, a function often used to run multiple tracking devices simultaneously for cross-validation of their respective performances [21], and an essential function for upgrade, maintenance, and quality control. Many leading research groups have adopted PLUS for developing image-guided intervention and therapy applications. 

OpenIGTLink [22] provides a unified solution to import real-time imagery (e.g., ultrasound, video) and general data streams (e.g., position tracking) into image-guided therapy and intervention systems. OpenIGTLink has become the default communication standard in its field. SlicerIGT, a dynamically loadable extension to 3D Slicer, enables the rapid translation of image-guided intervention and therapy applications without any programming overhead, using only configuration scripts [23]. Machine learning (ML) and artificial intelligence (AI) functions for training and invoking deep learning neural networks are grouped in the SlicerAIGT extension package. SlicerIGT offers great speed and ease for integrating new clinical applications and translating them to clinical trials. The generic theme of “spatially tracked ultrasound navigation” is an out-of-the-box feature which does not require any programming, and it works with most ultrasound and spatial tracking devices [23]. Building on this platform, the application-specific code, written as a Python script, is less than 0.01% of the code base, while the remaining 99.99% is free, open-source software infrastructure [23], allowing for an extremely high degree to which previously validated software can be reused. The functional and architectural designs of the NaviPBx system are complete. 

## 3. System Implementation

### 3.1. Preliminary Prototype

**Figure 3:**
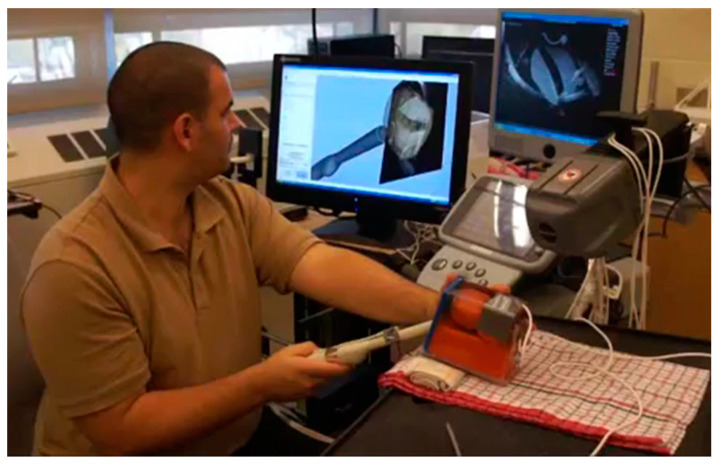
Preliminary prototype of the NaviPBx prostate biopsy system in a phantom setup.

System implementation has commenced. A preliminary prototype of NaviPBx is complete and is shown in Figure 3, in a training phantom setup. This prototype is currently using EM tracking which will be changed to optical tracking, as planned.

**Figure 4:**
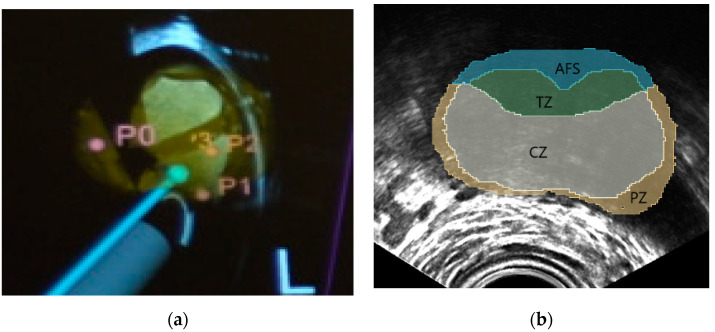
Typical system guidance views of the NaviPBx prostate biopsy system. (**a**) Models of the TRUS transducer (grey), biopsy needle (magenta), prostate gland (yellow), and the biopsy target locations (pink), that are overlaid on the live 2D TRUS image. (**b**) McNeal zones painted on the live 2D TRUS image.

NaviPBx guides the physician with real-time “labels” painted over the live 2D TRUS image, such as in Figure 4a, showing models of the TRUS transducer (grey), biopsy needle (magenta), prostate gland (yellow) and biopsy target locations (pink), that are overlaid on the live 2D TRUS image. The physician interfaces with NaviPBx through a lightweight user interface optimized to execute only the necessary workflow while hiding all other functions. The system records all intra-operative data (e.g., time-stamped ultrasound, tracker, video, audio, rendering scenes, user actions, and internal variables) for offline analysis and validation when the user interface is turned back on at full functionality.

### 3.2. Visualization of the Intraprostatic Anatomy

The visualization of the intraprostatic anatomy is currently under development, reusing several previously developed modules, as explained below. 

The prostate gland is the primary anatomical structure of interest, and automated segmentation of the gland is an essential function. We have previously demonstrated deep learning-based segmentation of the prostate in transrectal ultrasound [24]. We plan to reuse our previously developed implementation. Our current prototype involves predictions on 2D slices sampled radially around the approximate central axis of the prostate, followed by a reconstruction onto a 3D surface. A 2D U-Net is modified, trained, and validated on ground-truth labeled images from both end-firing and side-firing TRUS. Modifications of the standard U-Net include the addition of a 50% dropout during training and the use of transpose convolutions instead of standard upsampling, followed by convolution, to reduce overfitting and improve performance, respectively. This segmentation engine shows less than 1.0 mm mean surface distance error with respect to ground truth contours [25]. Considering the size of clinically significant prostate cancer, 0.5 cc in volume [11], this prostate segmentation prototype module is expected to achieve a clinically sufficient performance when fully integrated in NaviPBx. 

Labeling the McNeal zones (central, transition, peripheral and anterior zones) in TRUS is a critical function for planning and sampling the systematic biopsy target locations, typically 12 cores [7]. For this purpose, we plan to reuse our previously developed implementation. Our current prototype starts with a set of anonymized labeled prostate MRI volumes, with segmentations of the prostate and the McNeal zones. Having segmented the prostate in 3D TRUS, we select the MRI scan with the most similar prostate, based on statistical shape analysis, then perform a deformable elastic registration between the prostate glands in the selected MRI and the 3D TRUS, with an existing module (e.g., BRAINSFit) of 3D Slicer. Finally, we apply the resulting deformation field to map the McNeal zones onto the 3D TRUS, and then paint the McNeal zones on the live 2D TRUS image, such as in Figure 4b. In a preliminary experiment with seven expert urologists, this module correctly labeled the McNeal zones 92% of the time, mislabeling only the central zone where very few prostate cancers start [26]. We expect occasional mislabeling to disappear when we use a larger set of model-training MRI volumes, including a pool of patients with advanced disease. Over 80 anonymized labeled MRI/TRUS volume pairs are currently available to us for this purpose, from previous research. It must be noted that the most important aspect is the correct labeling of the McNeal zones, while the metric accuracy required for the zonal contours is quite lenient, considering the smallest size of significant prostate cancer to be biopsied, 0.5 cc in volume [11]. Altogether, this prostate anatomy labeling prototype module promises clinically sufficient performance when fully integrated in NaviPBx.

NaviPBx must maintain the anatomy labels accurately on the live 2D TRUS image, while the prostate is being displaced and deformed by rectal pressure from the TRUS transducer. For this purpose, we plan to reuse our previously developed implementation. Our protype exploits the practical observation that prostate displacement and deformation occurs mainly in the direction of the tracked TRUS probe. It uses an intensity-based algorithm with Powell optimization, initialized by tracking information and driven by the normalized cross-correlation metric [27]. The current protype yields a 1.6 mm mean registration accuracy over the whole prostate gland, with a 10 Hz update rate, thus promising a clinically sufficient performance when fully integrated into NaviPBx.

### 3.3. Visualization of Cancer-Suspicious Locations

The function used to visualize cancer-suspicious locations is explained as envisioned, as its implementation has not yet commenced. There is strong evidence in the peer-reviewed literature that multiparametric transrectal ultrasound (mpTRUS) imaging, specifically elasticity and Doppler imaging rendered on the B-mode TRUS image, are helpful adjuncts in PBx. Doppler ultrasound indicates a suspicion of focal neoplastic proliferation where the uncontrolled growth of blood vessels is observed in relation to the intraprostatic anatomy through the presentation of an increased number of tortuous and disordered vessels, while elasticity imaging assesses the tissue’s response to mechanical excitation by pressure or sonification force [10,28]. Abnormal color flow is strongly associated with high Gleason scores (8–10) while elastography has a positive association with Gleason scores of 5–10 [28] and a 75% detection rate for advanced PCa [29]; altogether very promising for Senegal, where over 60% of patients have an advanced form of the disease [12].

In current practice, the physician scans the prostate gland continuously, trying to mentally assemble and interpret the multiparametric information. To appreciate the extra information with respect to underlying intraprostatic anatomy is extremely difficult and time-consuming, especially because the intraprostatic anatomy is not visible. It further complicates the task that each TRUS image is unique and irreproducible; even if the physician manages to revisit the same acquisition plane twice, the resulting images are different due to subtle changes in probe pressure and acoustic coupling. Through real-time tracking of the transducer and the prostate gland, NaviPBx will compound a hyperdimensional array from the component modalities (B-mode, elasticity, and Doppler), allowing for the analysis of modalities together, in both 3D and 2D, using 3D Slicer’s extensive visualization arsenal. NaviPBx renders the resulting image as an overlay atop the intraprostatic anatomy labels. Moreover, in using standard B-mode TRUS, about 60% of PCa appears as hypoechogenic. As a starting observation, the physician looks for contiguous hypoechogenic areas in relation to the intraprostatic anatomy [10]. To aid in this task, NaviPBx will use a deep learning model to automatically segment contiguous hypoechogenic areas in the compound 3D volume by extending our prostate segmentation module [24,25]. We have over 80 labeled, anonymous TRUS volumes available for developing this function. We stress that NaviPBx will not suggest a clinical decision, but will render the component ultrasound modalities to augment the conventional images, consistent with how they were validated in prior peer-reviewed clinical literature.

Intensive research is underway by several leading groups on deep learning-based quantitative mpTRUS imaging toward identifying PCa [30]. The clinical utility and robustness of these methods is presently being studied. When a specific quantitative mpTRUS method matures, easy integration of deep learning models in the SlicerAIGI extension module will enable the addition of the new function without affecting workflow and safety. Deep learning predictions are computed locally, without pushing large data. One such emerging approach that we intend to pursue is Temporal-enhanced Ultrasound (TeUS), introduced by our team. 

TeUS achieves tissue typing by picking up subtle variations in a time series of radio-frequency ultrasound data acquired over a relatively short time span, using deep neural networks. TeUS was successfully demonstrated in ex vivo and in vivo experiments on 60 whole-mount prostate samples [31,32]. TeUS achieved superior PCa detection versus B-mode in 255 PBx cores from 158 patients [33]. Our analytical formulation describes the multiparametric relationship between TeUS and elasticity imaging for prostate tissue characterization [34]. This formulation is based on how TeUS captures the interplay between micromotions of the tissue microstructure and elasticity. In tissue types of similar mechanical properties, TeUS captures the spatial variations in the scattering function, which is expected to help detect PCa where changes in the nuclei configuration dominate in the tissue. In tissue types with differences in both mechanical and scattering properties, TeUS captures a combined effect, increasing sensitivity in detecting PCa. TeUS is currently in clinical evaluation in the USA and Canada. We will first evaluate these quantitative mpTRUS methods retrospectively by comparing their predictions with core tissue pathology before using them to treat patients.

### 3.4. Hardware Choices and Future Alternatives

By the time NaviPBx reaches the planned clinical deployment, there will have been generational changes in hardware and software technologies. We have designed NaviPBx to absorb such future changes with minimum modification, without hazarding the operational safety and performance of the system.

#### 3.4.1. Operating System

Our current choice of operating system and computing platform is a Windows tablet computer. Among the presently available Windows tablets, a Microsoft Surface Pro with 10th Gen Intel Core i5 CPU and 8 GB RAM is sufficient. It can produce fast 3D display updates and when an AI model is already trained, prediction is fast in the CPU. We do not need to add a GPU, neither for graphics, nor for the purpose of AI. The trained AI model can be updated at any time over internet or a USB stick; the file size of a trained AI model ranges from 10–20 MB. Alternatively, we could run the user interface on an inexpensive Windows tablet and mirror the image of a nearby high-performance desktop computer through WiFi. Desktop computers with the same specifications are generally less expensive and are easier to maintain, while the tablet only needs a screen sharing app installed once, without the need for update and maintenance.

Since 3D Slicer and all of its extensions run on all major operating systems without hardware dependency, the NaviPBx architecture is robust to any changes of operating systems and hardware. For the consistency of user experience, we will stay with Windows. We will continue to not use hardware-dependent accelerators, like GPUs; however, it is possible that emerging deep learning technologies require that we reconsider this stance.

#### 3.4.2. Tracking System

While the preliminary prototype works with the electromagnetic (EM) tracking option, for the clinical device the choice of tracking is OptiTrack™ Duo with a simultaneous infrared and video feed, from a leading provider (www.optitrack.com (accessed on 8 August 2021)). We will use flat infrared markers mounted on 3D-printed rigid reference frames, all produced in-house. Optical trackers are quickly becoming less expensive, more functional, and smaller. PLUS enables the upgrade to new trackers without risking safety, function, and accuracy. Current EM trackers are expensive and the technology is stagnant. There is a trend of increasingly performant position sensors embedded in TRUS probes. For example, the Clarius EC7 (www.clarius.com (accessed on 8 August 2021)), sports an integrated combo of a gyroscope, an accelerometer, and a magnetometer, which together provide exquisitely accurate orientation encoding. When the embedded tracking sensors can encode both position and orientation, we may dispense with external tracking, thereby significantly reducing the cost and complexity of NaviPBx.

#### 3.4.3. Ultrasound Hardware

Our current choice of US hardware is the handheld wireless software, Clarius EC7, which features the best-in-class image quality that is comparable to high-end cart systems, affordable pricing, color Doppler, and elasticity functions. Clarius has a software interface to scan controls (gain, focus, and acquisition trigger), radiofrequency data, and beam forming, and it supports the OpenIGTLink interface to PLUS. Clarius is a reputable company with strong support and worldwide distribution; its products are usable in Senegal under an import permit. Cloud storage is free with every Clarius scanner. We will explore this depending on how Senegalese laws allow for cloud storage of patient data, if they even allow for this possibility. The EC7 is compatible with the widely used CIVCO (www.civco.com (accessed on 8 August 2021)) clip-on needle guides. For maximum patient safety, we will use disposable needle guides, pre-sterilized by the vendor.

#### 3.4.4. Projected Costs of Production and Operation

While NaviPBx costs only a small fraction of the price of a commercial prostate biopsy navigation system, the price of production is still significant in the context of Senegal. The projected total cost of NaviPBx, as negotiated with vendors for the production of 15 systems, is USD 13,000, including Clarius EC7 TRUS with the requisite software options, OptiTrac™ Duo tracker, Windows tablet, a mounting rack, and a shockproof storage case. Altogether, the production cost of NaviPBx is about USD 2500 more than a conventional biopsy setup would be if using the same ultrasound system. We believe that the manifold benefits of spatiotemporal navigation are worth this difference in price. Furthermore, the trend of ever-reducing tracking hardware prices will make this difference even less significant in the future. The recurring operating expense above that of conventional biopsy is the cost of the clip-on needle guide, approximately USD 20 per patient at present. We expect that, in the long run, a domestic supplier will ultimately emerge in Senegal and significantly mitigate this cost.

## 4. Clinical Validation and Deployment Plan

### 4.1. Clinical Safety and Feasibility Study

Following an accuracy and workflow evaluation in training phantoms, we will first ensure the safety NaviPBx in human use through a Clinical Safety and Feasibility Study. We will implement a systematic risk management process following the ISO 14,971 standard. Additionally, technical accuracy validation, based on anatomical landmarks and recorded TRUS, will prove the ability to sample a clinically significant prostate cancer target, 0.5 cc in volume. This study will be performed at the Department of Urology of the Aristide Le Dantec University Hospital in Dakar, Senegal. The minimum number of patients will depend on whether major design revisions will be needed, currently estimated to be 50 patients over 1 year.

Research Ethics Approval will be obtained according to standing regulations in Senegal; the country has a nationally centralized process. The approval of a clinical study is valid in any government-acknowledged medical care facility in Senegal, which includes all clinical sites in this project: the Aristide Le Dantec University Hospital and all hospitals of the Senegalese Military Health Service.

We do not plan to analyze specific clinical outcomes in the early implementation phase of the program, for two reasons. Firstly, NaviPBx uses techniques that have been previously validated in peer-reviewed literature. Secondly, there is no reliable baseline data on PBx outcomes in Senegal, as the country’s PBx practice is limited and insufficiently documented. This project will yield nationwide PBx data to be analyzed in follow-up research. We will therefore concentrate our validation efforts on clinical usability and the scalability of nationwide deployment.

### 4.2. Formative Clinical Usability Study

Malfunctions caused by the inadequate usability of a medical device have become an increasing safety concern. Without thorough investigation of usability aspects, NaviPBx could be non-intuitive and difficult to learn and use, a critically important issue to be addressed before NaviPBx is passed into the hands of less experienced physicians. Formative usability testing will be performed to help developers identify design weaknesses. Formative testing is based on the expertise and knowledge of developers and how they interpret the user’s struggle with the system interface. Another aim of the formative test is to define a “minimum viable product” that incorporates the set of functions necessary and is sufficient for deploying a prospective system in clinical practice. We will implement a usability engineering process, according to the Risk Management Plan previously laid out, to identify and minimize erroneous use and reduce use-associated risks. Following the IEC 62,366 standard, the process will be executed in parallel to risk management. A first-order estimation for the formative usability study, following the guidelines in IEC 62,366 and Faulkner [35], suggests N = 5–10 urologists as users. The planned study locations are the Aristide Le Dantec University Hospital and the Military Hospitals of Ouakam, Principal and Lemonier, in Dakar, Senegal. Each hospital will participate with two to three attending urologists. The commander of the urological services in the Senegalese Military Health Service will coordinate with the military hospitals, with Ouakam Military Hospital in Dakar acting as the command center. For a military hospital to work efficiently, there is a threshold volume when the scale of economy starts to prevail. For the optimal utilization of the army’s logistical system, each hospital should carry a uniform load, so that the dispatching of personnel and materials can be uniformly managed from the command center. We estimate that 50 patients per year, per hospital would be the optimal intake. The projected length of participation is 1 year, enrolling about one patient per week and per hospital.

### 4.3. Summative Clinical Usability & Scalability Study

Finally, we will run a summative usability test in which users perform workflow tasks; performance is rated by the usability quotient computed for each design element previously identified in the Risk Management Plan. The summative test will identify the percentage of users having trouble performing a certain action. This will help us to modify the design and/or reimplement the problematic system elements. The second objective is it to evaluate how the operation of NaviPBx scales up to national use across the country, and to quantify the technical, material, and human resource requirements for operating the nationwide program. This information will be reviewed by the Senegalese Military Health Service, which intends to sustain the national program. Based on our previous experience, we estimate that 20–25 users are needed for a successful summative evaluation; this target can be achieved by involving two to three urologists from each of the participating hospitals, with corresponding nurses in each hospital. The targeted number of patients, for previously explained reasons related to the operation efficiency of the Senegalese Military Health Service, is 50 patients per year, per hospital. The planned study locations are 10 hospitals: the Aristide Le Dantec University Hospital; three military hospitals in Dakar; and one hospital in each military zone outside Dakar: Thies (mid-west), Saint-Louis (north), Ziguinchor and Kolda (south), Kaolack (central), and Tambacounda (east), covering the entire territory of Senegal. The projected project length is 2 years, enacted in two stages, each year with three new hospitals, enrolling approximately one patient per week and per hospital.

By end of the project, the clinical studies will have involved a combined 20–30 urologists (within 10 hospitals) and over 1000 patients, cumulatively.

### 4.4. Training Plan

We will establish mechanisms to train associated personnel (e.g., software engineers, technicians, nurses, physicians) who are qualified to partake in the proposed studies and who will sustain the operation of the prostate biopsy program. The curriculum will include instructional and practice sessions in simulation setup on training dummies and assist expert urologists in human patient cases performed with the NaviPBx system. The training plan also involves nurses’ and technicians’ participation in various tasks (e.g., preparation, calibration, setup, takedown, cleaning, transportation, and storage).

## 5. Project Timeline

The optimal planned timeline for completing the NaviBPx project is 5 years. At the time of writing this article, the project is in its first year. Functional and software architectural designs have been completed. Software implementation of the NaviBPx framework and several of the core functionalities are underway, but presently are still incomplete. By the end of the first year, we hope to complete the basic functional system, without the advanced functions of real-time visualization of the intraprostatic anatomy (discussed in Section 3.2) and visualization of cancer-suspicious locations (Section 3.3), and obtain research ethics approval for the first study on clinical experience. In the second year, we plan to complete the Clinical Safety and Feasibility Study (Section 4.1) and gradually supplement the prototype with technical developments towards a feature-complete NaviPBx system. We will bench test the system and update the research ethics approval as new features are added. In the third year, we aspire to usher NaviBPx through a Formative Clinical Usability Study (Section 4.2), and finally scale up the operation to a Summative Clinical Usability & Scalability Study in 10 hospitals across Senegal (Section 4.3).

## 6. Sustainability Plan

The Senegalese Military Health Service is committed to sustaining the NaviPBx program upon successful completion of the project. Senegal is one of the most stable countries in Africa, having experienced peaceful political transitions since its independence in 1960. The main mission of the Senegalese Military Medical Service is to provide medical support to the Armed Forces and ensure health protection at home and abroad, in peacetime and during operations. The Military Health Service has a longstanding civilian mandate within the Senegalese healthcare system, independently from political parties elected to government. Its facilities are open to the public, contributing to national public health coverage. In the care facilities operated by the Military Medical Service, over 90% of the patients are civilians with no connection to the military. The Service operates 14 military regional medical centers over the national territory, nine of which offer urological services and will partake in the NaviPBx program.

Our clinical studies aim to generate evidence of NaviPBx’s robust usability and scalability to nationwide deployment and to quantify the requisite technical, material, and human resources for the continued operation of the prostate biopsy program. The training plan for personnel (e.g., engineers, technicians, nurses, physicians), as outlined earlier, is a critical factor in the continual operation of the national prostate biopsy program.

Importantly, NaviPBx will be shared as a free, open-source software resource, and vigorously advocated as such. Given the successful history over the past two decades in promoting 3D Slicer platform resources, one can anticipate a robust uptake on NaviPBx as a diagnostic and therapy delivery platform for prostate cancer and beyond.

The commercialization of NaviPBx and/or its consumable parts in Senegal and/or regionally in West Africa is an option that is being actively explored, while noting that regulatory aspects of domestically produced medical devices are currently uncertain in Senegal.

We will promote the prostate biopsy program to the general public through our ongoing annual Prostate Disease Awareness Day (PDAD), organized by two expert urologists. A sweeping outreach effort in the Senegalese media and social platforms, PDAD aims to increase the awareness of prostate diseases, particularly of prostate cancer, and to inform patients about the role of prostate biopsy in the early diagnosis and curative management of prostate cancer. Participating media outlets include the Senegalese National Television, four private television networks, six radio stations, and two major social platforms. PDAD provides general information to the public and also free, walk-in urology consultation for ad hoc patients. In Dakar, an average of 55–60 physicians (urologists and general practitioners) participate, seeing 250 to 300 patients for clinical consultations. Upon launching NaviPBx in hospitals across the country, we will extend PDAD into a nationwide campaign. In a preliminary study [36], we found that using 3D digital anatomy models vastly improved the effectiveness of communication about prostate disease with patients in Senegal. Expanding on this idea, we will create video animations of NaviPBx for public release. Clinical stakeholders will receive project updates through newsletters and symposia of the Senegalese Urological Association, encouraging members to partake in our PDAD campaigns and to reach out to the public. In addition to the Senegalese Military Health Service, we will liaise with the Ministry of Health through demonstrations, regular progress reports, and by extending invitations to NaviPBx project events. The Société Internationale d’Urologie, the Association Francaise d’Urologie, and the American Urological Association will be regularly informed about our progress and results.

Building translational R&D capacities in Senegal is also a critical aspect of increasing the likelihood for the project to exert a sustained positive impact. We aim to help develop translational clinical research capacities in Senegal by training a large portion of Senegal’s urology staff in the Senegalese Military Health Service. Under the leadership of academically minded senior military commanders, this corps is expected to emerge as a highly organized, experienced, and productive translational research force. The clinical and engineering personnel emerging from this program stand to represent a national resource in Senegal, to be leveraged in research and in the development of image-guided diagnostic and therapeutic intervention technologies. The project personnel will gain experience in translational research and apply their knowledge to high-impact medical problems with practical and societal implications. Such a skillset will be of utmost value to the biotechnology and healthcare sectors in Senegal. A recent report by the World Economic Forum on the Future of Jobs identified emerging job roles in Sub-Saharan Africa through extensive surveys and studies of local industry. Amongst the top five emerging job roles were “Software Application Developers and Analysists, Data Analysts and Scientists,” with the critical skills of “analytical thinking and innovation, technology design and programing” [37]. Overall, the diverse pool of individuals trained through this project will be important stakeholders in the emergent healthcare job market in Senegal.

## 7. Conclusions

We presented the design and ongoing development of a PBx system and program for nationwide deployment in Senegal. The Senegalese Military Health Service is committed to sustaining the program upon successful initial implementation. Through this program, we also aspire to help develop translational clinical research capacities in Senegal. The free open-source technology and highly trained clinical and engineering personnel emerging from this program will represent a national resource for Senegal, to be leveraged by time-efficient development and large-scale implementation of further image-guided intervention programs.

## Data Availability

Anonymized patient image data used for development of the system can be made available upon request, subject to an institutional research agreement. Software modules used in the current implementation are available as free open source under www.slicer.org (accessed on 8 August 2021).
